# Crystal structure of 3-amino-1-(4-hy­droxy­phen­yl)-1*H*-benzo[*f*]chromene-2-carbo­nitrile

**DOI:** 10.1107/S2056989015012566

**Published:** 2015-07-04

**Authors:** Mehmet Akkurt, Peter N. Horton, Sabry H. H. Younes, Shaaban K. Mohamed, Mustafa R. Albayati

**Affiliations:** a Department of Physics, Faculty of Sciences, Erciyes University, 38039 Kayseri, Turkey; b School of Chemistry, University of Southampton, Highfield, Southampton SO17 1BJ, England; c Chemistry Department, Faculty of Science, Sohag University, 82524 Sohag, Egypt; d Chemistry and Environmental Division, Manchester Metropolitan University, Manchester M1 5GD, England; e Chemistry Department, Faculty of Science, Minia University, 61519 El-Minia, Egypt; f Kirkuk University, College of Science, Department of Chemistry, Kirkuk, Iraq

**Keywords:** crystal structure, amino­chromenes, hydrogen bonding, N—H⋯π(arene) inter­actions

## Abstract

In the title compound, C_20_H_14_N_2_O_2_, the hy­droxy­benzene ring is almost perpendicular to the mean plane of the naphthalene ring system, making a dihedral angle of 85.56 (4)°. The 4*H*-pyran ring fused with the naphthalene ring system has a flattened boat conformation. In the crystal, O—H⋯N and N—H⋯O hydrogen bonds link the mol­ecules into a supra­molecular layer in the *bc* plane; N—H⋯π inter­actions also contribute to this arrangement. The layers are linked by weak by C—H⋯π and π–π [inter-centroid separation = 3.8713 (7) Å] inter­actions.

## Related literature   

For the biological activity of some heterocyclic derivatives containing the 4*H*-pyran unit, see: Elnagdi *et al.* (1983 ▸); Goldmann & Stoltefus (1991 ▸); Perez-Perez *et al.* (1995 ▸); Fan *et al.* (2010 ▸); Aytemir *et al.* (2004 ▸); Uher *et al.* (1994 ▸). For similar structures, see: Akkurt *et al.* (2013 ▸, 2015 ▸).
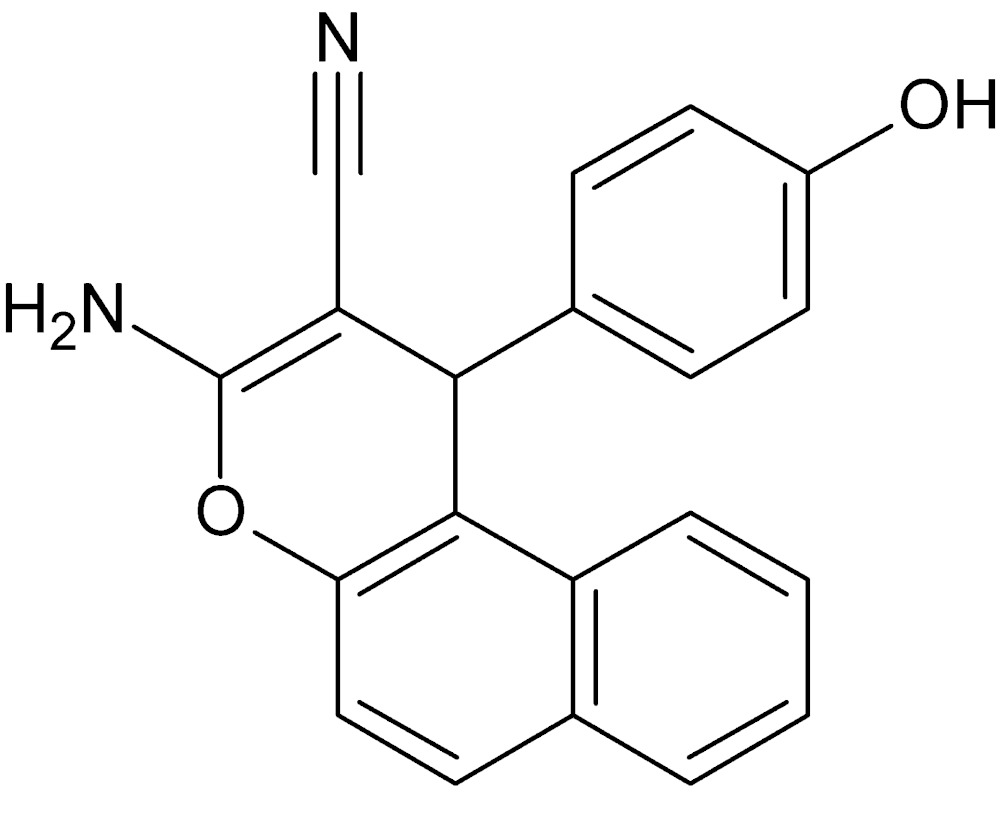


## Experimental   

### Crystal data  

C_20_H_14_N_2_O_2_
*M* *_r_* = 314.33
Monoclinic, 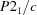

*a* = 12.1086 (8) Å
*b* = 13.1418 (9) Å
*c* = 10.1552 (7) Å
β = 96.992 (1)°
*V* = 1603.97 (19) Å^3^
*Z* = 4
Mo *K*α radiation
μ = 0.09 mm^−1^
*T* = 100 K
0.21 × 0.08 × 0.07 mm

### Data collection  

Rigaku AFC12 (Right) diffractometer
Absorption correction: multi-scan (*CrystalClear-SM Expert*; Rigaku, 2012 ▸) *T*_min_ = 0.738, *T*_max_ = 1.000
17411 measured reflections
3679 independent reflections
3251 reflections with *I* > 2σ(*I*)
*R*_int_ = 0.030

### Refinement  

*R*[*F* ^2^ > 2σ(*F* ^2^)] = 0.034
*wR*(*F* ^2^) = 0.092
*S* = 1.06
3679 reflections
229 parameters
3 restraints
H atoms treated by a mixture of independent and constrained refinement
Δρ_max_ = 0.26 e Å^−3^
Δρ_min_ = −0.18 e Å^−3^

### 

Data collection: *CrystalClear-SM Expert* (Rigaku, 2012 ▸); cell refinement: *CrystalClear-SM Expert*; data reduction: *CrystalClear-SM Expert*; program(s) used to solve structure: *SHELXS97* (Sheldrick, 2008 ▸); program(s) used to refine structure: *SHELXL2012* (Sheldrick, 2015 ▸); molecular graphics: *ORTEP-3 for Windows* (Farrugia, 2012 ▸); software used to prepare material for publication: *WinGX* (Farrugia, 2012 ▸).

## Supplementary Material

Crystal structure: contains datablock(s) I. DOI: 10.1107/S2056989015012566/tk5372sup1.cif

Structure factors: contains datablock(s) I. DOI: 10.1107/S2056989015012566/tk5372Isup2.hkl

Supporting information file. DOI: 10.1107/S2056989015012566/tk5372Isup3.cml

**Fig. 1.**
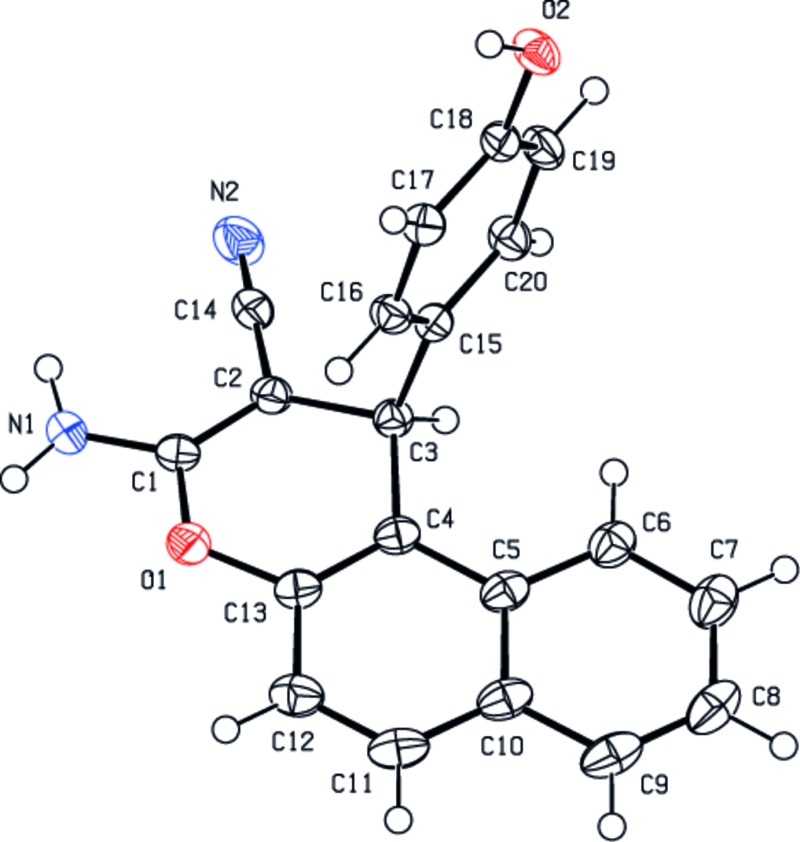
View of the title compound with the atom-numbering scheme. Displacement ellipsoids for non-H atoms are drawn at the 50% probability level.

**Fig. 2.**
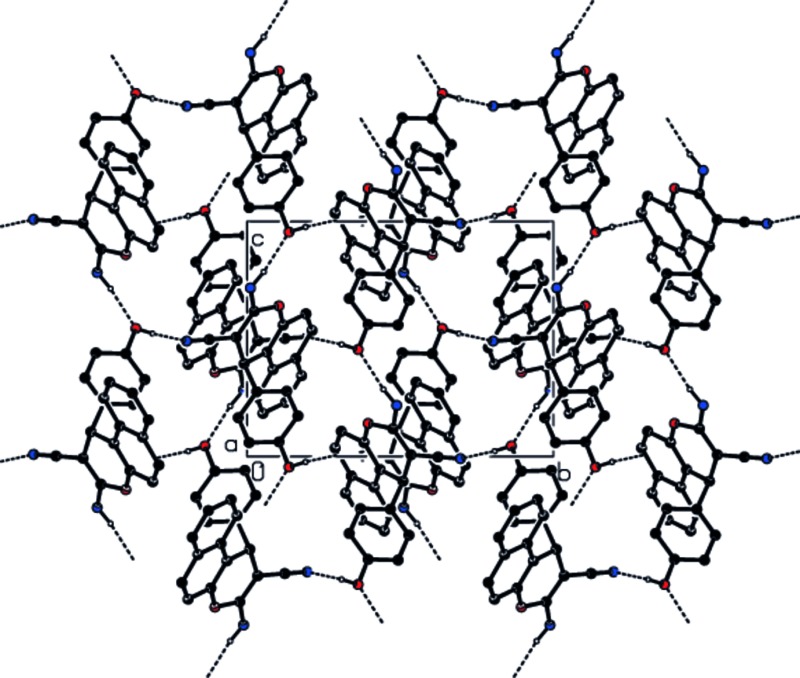
View of the packing of the title compound viewing down a axis.

. DOI: 10.1107/S2056989015012566/tk5372fig1.tif
View of the title compound with the atom-numbering scheme. Displacement ellipsoids for non-H atoms are drawn at the 50% probability level.

a . DOI: 10.1107/S2056989015012566/tk5372fig2.tif
View of the packing of the title compound viewing down *a* axis.

CCDC reference: 1409621

Additional supporting information:  crystallographic information; 3D view; checkCIF report

## Figures and Tables

**Table 1:** Hydrogen-bond geometry (, ) *Cg*3 and *Cg*4 are the centroids of the C5C10 and C15C20 rings, respectively.

*D*H*A*	*D*H	H*A*	*D* *A*	*D*H*A*
N1H1*N*O2^i^	0.90(1)	2.03(1)	2.9191(12)	173(1)
O2H2*O*N2^ii^	0.89(1)	1.86(1)	2.7403(12)	175(2)
N1H2*N* *Cg*4^iii^	0.88(1)	2.55(1)	3.2340(11)	135(1)
C11H11*Cg*3^iv^	0.95	2.97	3.7610(13)	142

Symmetry codes: (i) 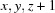; (ii) 
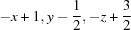
; (iii) 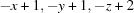; (iv) 
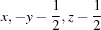
.

## supplementary crystallographic information

### S1. Comment

During the last decade, 4*H*-pyrans have held a unique role in medicinal chemistry due to their biological and pharmacological activities (Elnagdi *et al.*, 1983; Goldmann & Stoltefus, 1991). Fused pyran derivatives also exhibit a wide spectrum of biological and pharmacological properties, such as antiviral and antileishmanial (Perez-Perez *et al.*, 1995; Fan *et al.*, 2010), anticonvulsant and antimicrobial (Aytemir *et al.*, 2004) and insecticidal (Uher *et al.*, 1994). In this context we report in this study the synthesis and crystal structural determination for the title compound.

In the title compound (Fig. 1), the hydroxy-benzene ring (C15–C20) is approximately perpendicular to the naphthalene ring system [C4–C13, maximum deviation = -0.017 (1) Å at atom C13] as indicated by the dihedral angle of 85.56 (4)°. The 4*H*-pyran ring (O1/C1–C4/C13) in the title compound is puckered with the puckering parameters of Q_T_ = 0.199 (1) Å, θ = 102.9 (3) ° and φ = 354.2 (3) °. The bond lengths and angles in the title compound are within normal ranges and comparable with those reported for the similar structures (Akkurt *et al.*, 2013; 2015).

In the crystal structure, molecules are linked by N—H···O and O—H···N hydrogen bonds (Table 1, Fig. 2), which leads to a layer in the *bc* plane.

### S2. Experimental

The title compound was obtained in 95% yield from the reaction of 2-naphthol (144 mg; 1 mmol) and an equimolar amount of 4-hydroxybenzylidene-malononitrile (180 mg; 1 mmol) in absolute ethanol (10 ml ) in the presence of a catalytic amount of piperidine under reflux for 3 h. Crystallization of the crude product from ethanol gave colourless crystals of the title compound suitable for X-ray crystallography·M.pt: 521 K.

### S3. Refinement

The H atoms of the OH and NH_2_ group were located in a difference Fourier map and were refined freely [N1—H1N = 0.896 (12) Å, N1—H2N = 0.883 (12) Å and O2—H2O = 0.885 (13) Å]. The H atoms attached to the C atoms were positioned geometrically, with C—H = 0.95 Å and C—H = 1.00 Å for aromatic and methine H, respectively, and with *U*_iso_(H) = 1.2*U*_eq_(C).

### Crystal data

**Table:**

C_20_H_14_N_2_O_2_	*F*(000) = 656
*M**_r_* = 314.33	*D*_x_ = 1.302 Mg m^−^^3^
Monoclinic, *P*2_1_/*c*	Mo *K*α radiation, λ = 0.71075 Å
*a* = 12.1086 (8) Å	Cell parameters from 17749 reflections
*b* = 13.1418 (9) Å	θ = 2.3–27.5°
*c* = 10.1552 (7) Å	µ = 0.09 mm^−^^1^
β = 96.992 (1)°	*T* = 100 K
*V* = 1603.97 (19) Å^3^	Blade, colourless
*Z* = 4	0.21 × 0.08 × 0.07 mm

### Data collection

**Table:**

Rigaku AFC12 (Right) diffractometer	3679 independent reflections
Radiation source: Rotating Anode	3251 reflections with *I* > 2σ(*I*)
Detector resolution: 28.5714 pixels mm^-1^	*R*_int_ = 0.030
profile data from ω–scans	θ_max_ = 27.5°, θ_min_ = 2.9°
Absorption correction: multi-scan (*CrystalClear-SM Expert*; Rigaku, 2012)	*h* = −15→15
*T*_min_ = 0.738, *T*_max_ = 1.000	*k* = −17→14
17411 measured reflections	*l* = −12→13

### Refinement

**Table:**

Refinement on *F*^2^	3 restraints
Least-squares matrix: full	Hydrogen site location: mixed
*R*[*F*^2^ > 2σ(*F*^2^)] = 0.034	H atoms treated by a mixture of independent and constrained refinement
*wR*(*F*^2^) = 0.092	*w* = 1/[σ^2^(*F*_o_^2^) + (0.0425*P*)^2^ + 0.4007*P*] where *P* = (*F*_o_^2^ + 2*F*_c_^2^)/3
*S* = 1.06	(Δ/σ)_max_ = 0.001
3679 reflections	Δρ_max_ = 0.26 e Å^−^^3^
229 parameters	Δρ_min_ = −0.18 e Å^−^^3^

### Special details

**Table:**

Geometry. All e.s.d.'s (except the e.s.d. in the dihedral angle between two l.s. planes) are estimated using the full covariance matrix. The cell e.s.d.'s are taken into account individually in the estimation of e.s.d.'s in distances, angles and torsion angles; correlations between e.s.d.'s in cell parameters are only used when they are defined by crystal symmetry. An approximate (isotropic) treatment of cell e.s.d.'s is used for estimating e.s.d.'s involving l.s. planes.

### Fractional atomic coordinates and isotropic or equivalent isotropic displacement parameters (Å^2^)

**Table:**

	*x*	*y*	*z*	*U*_iso_*/*U*_eq_	
C1	0.33983 (8)	0.47639 (8)	1.11802 (10)	0.0237 (2)	
C2	0.31777 (8)	0.53512 (7)	1.00751 (9)	0.0225 (2)	
C3	0.22804 (8)	0.50920 (7)	0.89492 (9)	0.02158 (19)	
H3	0.1865	0.5730	0.8672	0.026*	
C4	0.14741 (8)	0.43453 (7)	0.94430 (10)	0.0234 (2)	
C5	0.04093 (8)	0.41495 (8)	0.87013 (10)	0.0261 (2)	
C6	0.00288 (9)	0.46750 (8)	0.75126 (11)	0.0295 (2)	
H6	0.0494	0.5170	0.7178	0.035*	
C7	−0.10048 (9)	0.44795 (9)	0.68361 (12)	0.0356 (3)	
H7	−0.1250	0.4847	0.6049	0.043*	
C8	−0.17001 (9)	0.37399 (10)	0.73009 (13)	0.0380 (3)	
H8	−0.2408	0.3603	0.6822	0.046*	
C9	−0.13586 (9)	0.32188 (9)	0.84389 (13)	0.0363 (3)	
H9	−0.1836	0.2722	0.8747	0.044*	
C10	−0.03025 (9)	0.34047 (8)	0.91728 (11)	0.0300 (2)	
C11	0.00527 (9)	0.28765 (9)	1.03649 (12)	0.0348 (3)	
H11	−0.0417	0.2374	1.0675	0.042*	
C12	0.10573 (9)	0.30770 (8)	1.10727 (12)	0.0325 (2)	
H12	0.1287	0.2723	1.1875	0.039*	
C13	0.17513 (8)	0.38188 (8)	1.05958 (10)	0.0256 (2)	
C14	0.38247 (9)	0.62331 (8)	0.99791 (10)	0.0264 (2)	
C15	0.27956 (8)	0.46801 (7)	0.77552 (9)	0.02106 (19)	
C16	0.32470 (8)	0.37029 (7)	0.77790 (10)	0.0233 (2)	
H16	0.3201	0.3280	0.8529	0.028*	
C17	0.37626 (8)	0.33360 (7)	0.67264 (10)	0.0237 (2)	
H17	0.4067	0.2669	0.6758	0.028*	
C18	0.38308 (8)	0.39533 (8)	0.56223 (9)	0.0235 (2)	
C19	0.33979 (9)	0.49308 (8)	0.55907 (10)	0.0272 (2)	
H19	0.3453	0.5357	0.4846	0.033*	
C20	0.28830 (8)	0.52852 (8)	0.66516 (10)	0.0255 (2)	
H20	0.2585	0.5955	0.6622	0.031*	
N1	0.42225 (8)	0.48875 (8)	1.21728 (9)	0.0297 (2)	
N2	0.43322 (9)	0.69661 (8)	0.99112 (10)	0.0383 (2)	
O1	0.27537 (6)	0.39555 (6)	1.14083 (7)	0.02776 (17)	
O2	0.43199 (7)	0.36270 (6)	0.45510 (7)	0.02998 (18)	
H1N	0.4259 (12)	0.4453 (10)	1.2856 (13)	0.042 (4)*	
H2N	0.4767 (11)	0.5317 (10)	1.2069 (14)	0.041 (4)*	
H2O	0.4737 (13)	0.3086 (11)	0.4770 (16)	0.057 (5)*	

### Atomic displacement parameters (Å^2^)

**Table:**

	*U*^11^	*U*^22^	*U*^33^	*U*^12^	*U*^13^	*U*^23^
C1	0.0230 (5)	0.0260 (5)	0.0232 (5)	0.0009 (4)	0.0073 (4)	−0.0010 (4)
C2	0.0224 (5)	0.0251 (5)	0.0207 (4)	−0.0014 (4)	0.0050 (3)	−0.0007 (4)
C3	0.0210 (4)	0.0223 (4)	0.0217 (4)	0.0009 (3)	0.0037 (3)	0.0003 (3)
C4	0.0221 (5)	0.0226 (5)	0.0266 (5)	0.0006 (4)	0.0074 (4)	−0.0028 (4)
C5	0.0226 (5)	0.0257 (5)	0.0312 (5)	0.0012 (4)	0.0080 (4)	−0.0073 (4)
C6	0.0246 (5)	0.0322 (5)	0.0318 (5)	0.0016 (4)	0.0040 (4)	−0.0067 (4)
C7	0.0281 (5)	0.0410 (6)	0.0370 (6)	0.0051 (5)	0.0009 (4)	−0.0108 (5)
C8	0.0223 (5)	0.0430 (7)	0.0483 (7)	0.0012 (5)	0.0022 (5)	−0.0184 (5)
C9	0.0251 (5)	0.0330 (6)	0.0529 (7)	−0.0045 (4)	0.0130 (5)	−0.0159 (5)
C10	0.0249 (5)	0.0266 (5)	0.0405 (6)	−0.0014 (4)	0.0116 (4)	−0.0089 (4)
C11	0.0317 (5)	0.0279 (5)	0.0477 (7)	−0.0057 (4)	0.0164 (5)	−0.0007 (5)
C12	0.0338 (6)	0.0287 (5)	0.0370 (6)	−0.0008 (4)	0.0119 (5)	0.0051 (4)
C13	0.0237 (5)	0.0250 (5)	0.0293 (5)	0.0004 (4)	0.0075 (4)	−0.0004 (4)
C14	0.0277 (5)	0.0328 (5)	0.0189 (4)	−0.0026 (4)	0.0035 (4)	0.0001 (4)
C15	0.0190 (4)	0.0237 (5)	0.0204 (4)	−0.0002 (3)	0.0020 (3)	−0.0005 (3)
C16	0.0251 (5)	0.0232 (5)	0.0221 (4)	−0.0006 (4)	0.0046 (4)	0.0033 (4)
C17	0.0251 (5)	0.0206 (4)	0.0258 (5)	0.0017 (4)	0.0045 (4)	0.0009 (4)
C18	0.0224 (4)	0.0285 (5)	0.0196 (4)	0.0012 (4)	0.0032 (3)	−0.0011 (4)
C19	0.0308 (5)	0.0294 (5)	0.0215 (5)	0.0051 (4)	0.0045 (4)	0.0067 (4)
C20	0.0279 (5)	0.0238 (5)	0.0249 (5)	0.0058 (4)	0.0039 (4)	0.0033 (4)
N1	0.0284 (5)	0.0386 (5)	0.0218 (4)	−0.0026 (4)	0.0020 (3)	0.0042 (4)
N2	0.0448 (6)	0.0404 (6)	0.0292 (5)	−0.0161 (5)	0.0028 (4)	0.0030 (4)
O1	0.0269 (4)	0.0280 (4)	0.0285 (4)	−0.0009 (3)	0.0036 (3)	0.0063 (3)
O2	0.0364 (4)	0.0326 (4)	0.0223 (4)	0.0095 (3)	0.0091 (3)	0.0022 (3)

### Geometric parameters (Å, º)

**Table:**

C1—N1	1.3393 (13)	C11—C12	1.3612 (17)
C1—O1	1.3548 (12)	C11—H11	0.9500
C1—C2	1.3614 (14)	C12—C13	1.4108 (14)
C2—C14	1.4089 (14)	C12—H12	0.9500
C2—C3	1.5171 (13)	C13—O1	1.3937 (12)
C3—C4	1.5122 (13)	C14—N2	1.1492 (14)
C3—C15	1.5286 (13)	C15—C20	1.3887 (13)
C3—H3	1.0000	C15—C16	1.3948 (13)
C4—C13	1.3661 (14)	C16—C17	1.3883 (13)
C4—C5	1.4351 (14)	C16—H16	0.9500
C5—C6	1.4180 (15)	C17—C18	1.3945 (13)
C5—C10	1.4250 (15)	C17—H17	0.9500
C6—C7	1.3765 (15)	C18—O2	1.3693 (11)
C6—H6	0.9500	C18—C19	1.3864 (14)
C7—C8	1.4047 (18)	C19—C20	1.3892 (14)
C7—H7	0.9500	C19—H19	0.9500
C8—C9	1.3636 (19)	C20—H20	0.9500
C8—H8	0.9500	N1—H1N	0.896 (12)
C9—C10	1.4207 (15)	N1—H2N	0.883 (12)
C9—H9	0.9500	O2—H2O	0.885 (13)
C10—C11	1.4162 (17)		
			
N1—C1—O1	111.01 (9)	C12—C11—C10	121.03 (10)
N1—C1—C2	127.23 (10)	C12—C11—H11	119.5
O1—C1—C2	121.75 (9)	C10—C11—H11	119.5
C1—C2—C14	117.94 (9)	C11—C12—C13	118.93 (10)
C1—C2—C3	122.99 (9)	C11—C12—H12	120.5
C14—C2—C3	119.07 (8)	C13—C12—H12	120.5
C4—C3—C2	109.14 (8)	C4—C13—O1	123.20 (9)
C4—C3—C15	112.06 (8)	C4—C13—C12	123.35 (10)
C2—C3—C15	110.69 (8)	O1—C13—C12	113.46 (9)
C4—C3—H3	108.3	N2—C14—C2	178.35 (12)
C2—C3—H3	108.3	C20—C15—C16	118.23 (9)
C15—C3—H3	108.3	C20—C15—C3	121.06 (8)
C13—C4—C5	118.06 (9)	C16—C15—C3	120.61 (8)
C13—C4—C3	120.77 (9)	C17—C16—C15	121.23 (9)
C5—C4—C3	121.16 (9)	C17—C16—H16	119.4
C6—C5—C10	118.25 (10)	C15—C16—H16	119.4
C6—C5—C4	122.52 (10)	C16—C17—C18	119.55 (9)
C10—C5—C4	119.23 (10)	C16—C17—H17	120.2
C7—C6—C5	121.03 (11)	C18—C17—H17	120.2
C7—C6—H6	119.5	O2—C18—C19	118.08 (9)
C5—C6—H6	119.5	O2—C18—C17	122.03 (9)
C6—C7—C8	120.47 (12)	C19—C18—C17	119.90 (9)
C6—C7—H7	119.8	C18—C19—C20	119.79 (9)
C8—C7—H7	119.8	C18—C19—H19	120.1
C9—C8—C7	120.00 (11)	C20—C19—H19	120.1
C9—C8—H8	120.0	C15—C20—C19	121.29 (9)
C7—C8—H8	120.0	C15—C20—H20	119.4
C8—C9—C10	121.26 (11)	C19—C20—H20	119.4
C8—C9—H9	119.4	C1—N1—H1N	117.9 (9)
C10—C9—H9	119.4	C1—N1—H2N	119.1 (9)
C11—C10—C9	121.63 (10)	H1N—N1—H2N	122.1 (13)
C11—C10—C5	119.39 (10)	C1—O1—C13	118.57 (8)
C9—C10—C5	118.98 (11)	C18—O2—H2O	109.9 (11)
			
N1—C1—C2—C14	−4.87 (16)	C9—C10—C11—C12	−178.64 (10)
O1—C1—C2—C14	173.97 (9)	C5—C10—C11—C12	0.69 (16)
N1—C1—C2—C3	174.55 (9)	C10—C11—C12—C13	−0.47 (17)
O1—C1—C2—C3	−6.61 (15)	C5—C4—C13—O1	−179.02 (9)
C1—C2—C3—C4	18.81 (13)	C3—C4—C13—O1	2.51 (15)
C14—C2—C3—C4	−161.78 (9)	C5—C4—C13—C12	1.68 (15)
C1—C2—C3—C15	−104.97 (10)	C3—C4—C13—C12	−176.79 (9)
C14—C2—C3—C15	74.44 (11)	C11—C12—C13—C4	−0.76 (16)
C2—C3—C4—C13	−16.41 (12)	C11—C12—C13—O1	179.88 (9)
C15—C3—C4—C13	106.56 (10)	C4—C3—C15—C20	134.98 (10)
C2—C3—C4—C5	165.16 (8)	C2—C3—C15—C20	−102.93 (10)
C15—C3—C4—C5	−71.87 (11)	C4—C3—C15—C16	−48.65 (12)
C13—C4—C5—C6	178.20 (9)	C2—C3—C15—C16	73.44 (11)
C3—C4—C5—C6	−3.33 (14)	C20—C15—C16—C17	−0.56 (14)
C13—C4—C5—C10	−1.40 (14)	C3—C15—C16—C17	−177.04 (9)
C3—C4—C5—C10	177.07 (9)	C15—C16—C17—C18	−0.08 (15)
C10—C5—C6—C7	0.61 (15)	C16—C17—C18—O2	−179.34 (9)
C4—C5—C6—C7	−178.99 (10)	C16—C17—C18—C19	0.85 (15)
C5—C6—C7—C8	−1.03 (16)	O2—C18—C19—C20	179.21 (9)
C6—C7—C8—C9	0.84 (17)	C17—C18—C19—C20	−0.97 (16)
C7—C8—C9—C10	−0.24 (17)	C16—C15—C20—C19	0.45 (15)
C8—C9—C10—C11	179.16 (10)	C3—C15—C20—C19	176.91 (9)
C8—C9—C10—C5	−0.17 (16)	C18—C19—C20—C15	0.31 (16)
C6—C5—C10—C11	−179.35 (9)	N1—C1—O1—C13	169.17 (8)
C4—C5—C10—C11	0.26 (14)	C2—C1—O1—C13	−9.84 (14)
C6—C5—C10—C9	−0.01 (14)	C4—C13—O1—C1	12.00 (14)
C4—C5—C10—C9	179.60 (9)	C12—C13—O1—C1	−168.63 (9)

### Hydrogen-bond geometry (Å, º)

Cg3 and Cg4 are the centroids of the C5–C10 and C15–C20 rings, respectively.

**Table:**

*D*—H···*A*	*D*—H	H···*A*	*D*···*A*	*D*—H···*A*
N1—H1*N*···O2^i^	0.90 (1)	2.03 (1)	2.9191 (12)	173 (1)
O2—H2*O*···N2^ii^	0.89 (1)	1.86 (1)	2.7403 (12)	175 (2)
N1—H2*N*···*Cg*4^iii^	0.88 (1)	2.55 (1)	3.2340 (11)	135 (1)
C11—H11···*Cg*3^iv^	0.95	2.97	3.7610 (13)	142

Symmetry codes: (i) *x*, *y*, *z*+1; (ii) −*x*+1, *y*−1/2, −*z*+3/2; (iii) −*x*+1, −*y*+1, −*z*+2; (iv) *x*, −*y*−1/2, *z*−1/2.
