# Supplementary figures and images for: Crystal structure of 3-amino-1-(4-hy­droxy­phen­yl)-1H-benzo[f]chromene-2-carbo­nitrile
Source: Acta Crystallogr E Crystallogr Commun. 2015 Jul 4;71(Pt 8):o536–7. doi: 10.1107/S2056989015012566 (PMC4571384; doi:10.1107/S2056989015012566)

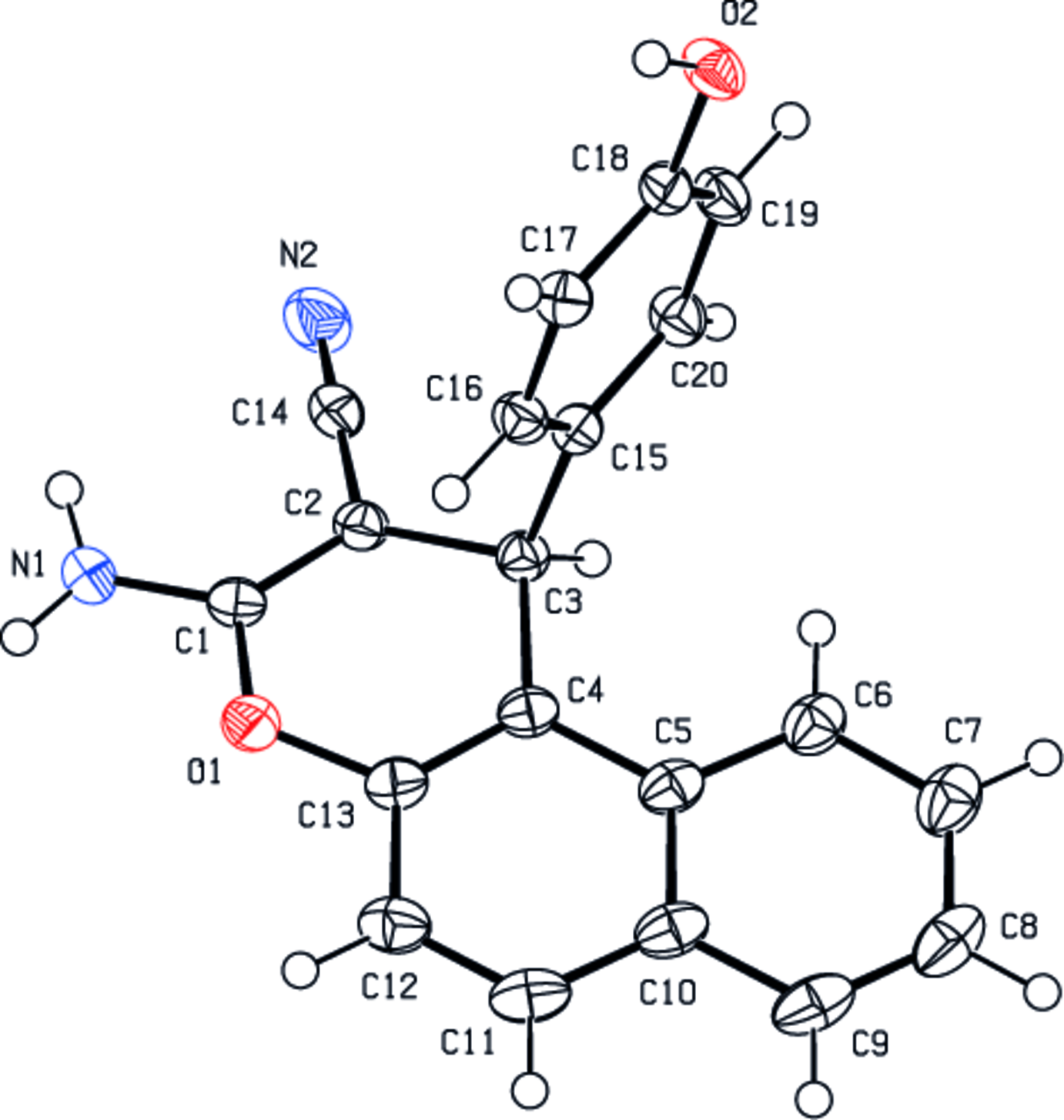

Supplement: Supplementary file 4 [file e-71-0o536-fig1.tif]

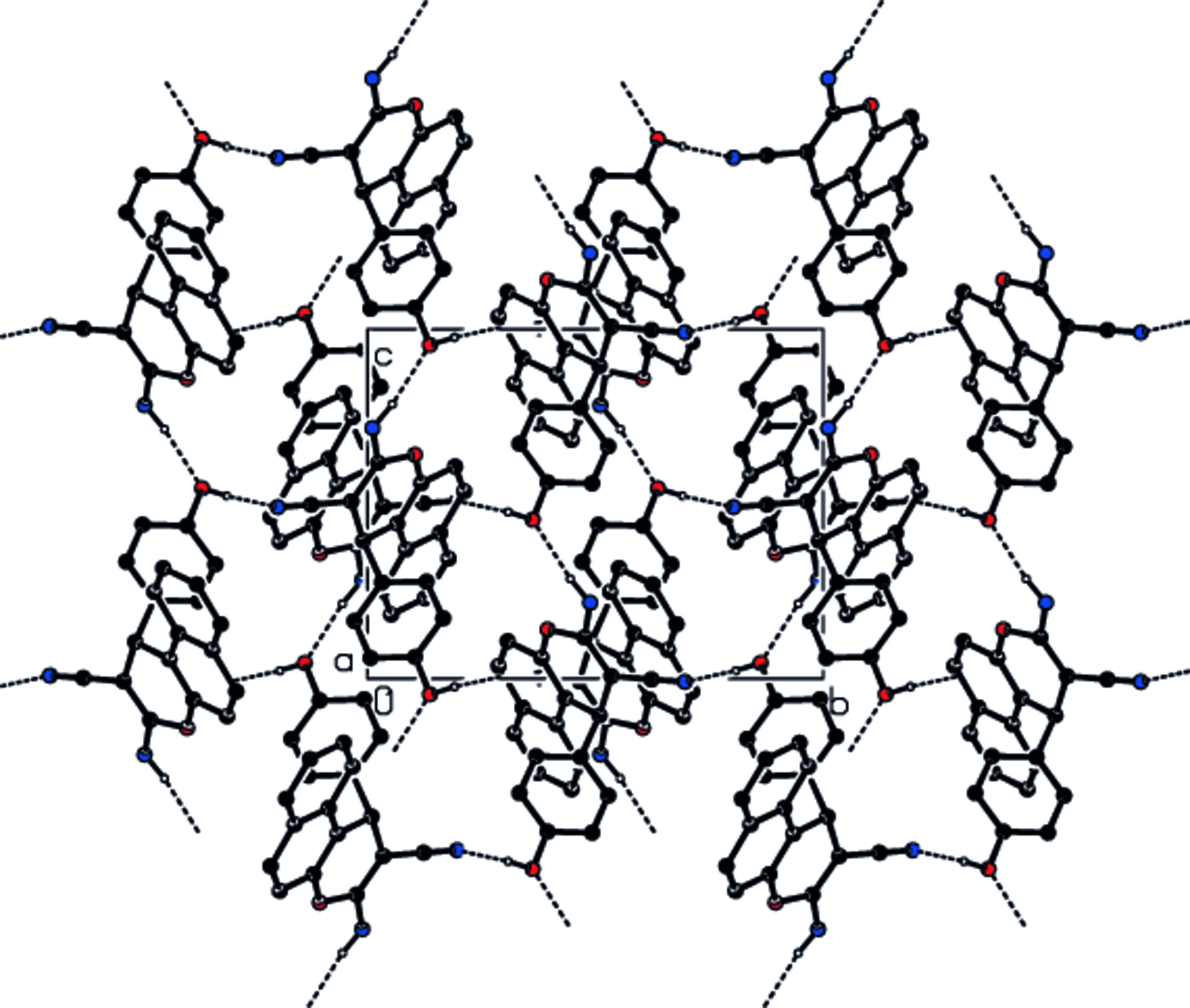

Supplement: Supplementary file 5 [file e-71-0o536-fig2.tif]
